# Ensemble machine learning reveals key features for diabetes duration from electronic health records

**DOI:** 10.7717/peerj-cs.1896

**Published:** 2024-02-26

**Authors:** Gabriel Cerono, Davide Chicco

**Affiliations:** 1 Department of Neurology, University of California San Francisco, San Francisco, CA, USA; 2 Institute of Health Policy Management and Evaluation, University of Toronto, Toronto, Canada; 3 Dipartimento di Informatica Sistemistica e Comunicazione, Università di Milano-Bicocca, Milan, Italy

**Keywords:** Diabetes, Diabetes type 1, Supervised machine learning, Data mining, Electronic health records, Health informatics, Medical informatics, Diabetes type 2, Feature ranking

## Abstract

Diabetes is a metabolic disorder that affects more than 420 million of people worldwide, and it is caused by the presence of a high level of sugar in blood for a long period. Diabetes can have serious long-term health consequences, such as cardiovascular diseases, strokes, chronic kidney diseases, foot ulcers, retinopathy, and others. Even if common, this disease is uneasy to spot, because it often comes with no symptoms. Especially for diabetes type 2, that happens mainly in the adults, knowing how long the diabetes has been present for a patient can have a strong impact on the treatment they can receive. This information, although pivotal, might be absent: for some patients, in fact, the year when they received the diabetes diagnosis might be well-known, but the year of the disease unset might be unknown. In this context, machine learning applied to electronic health records can be an effective tool to predict the past duration of diabetes for a patient. In this study, we applied a regression analysis based on several computational intelligence methods to a dataset of electronic health records of 73 patients with diabetes type 1 with 20 variables and another dataset of records of 400 patients of diabetes type 2 with 49 variables. Among the algorithms applied, Random Forests was able to outperform the other ones and to efficiently predict diabetes duration for both the cohorts, with the regression performances measured through the coefficient of determination R^2^. Afterwards, we applied the same method for feature ranking, and we detected the most relevant factors of the clinical records correlated with past diabetes duration: age, insulin intake, and body-mass index. Our study discoveries can have profound impact on clinical practice: when the information about the duration of diabetes of patient is missing, medical doctors can use our tool and focus on age, insulin intake, and body-mass index to infer this important aspect. Regarding limitations, unfortunately we were unable to find additional dataset of EHRs of patients with diabetes having the same variables of the two analyzed here, so we could not verify our findings on a validation cohort.

## Introduction

Diabetes mellitus is group of metabolic diseases characterized by hyperglycemia and an epidemic affecting more than 420 million of people worldwide (Chatterjee, Khunti & Davies, 2017). Diabetes mellitus can be classified in two main types: type 1 (T1DM) and type 2 (T2DM). T2DM often occurs in older populations, accounting for 90% of total diabetes cases (Sattar et al., 2019), although it is increasingly seen in younger people (Chen, Magliano & Zimmet, 2012). T2DM appears with a gradual onset and is characterized by an impaired insulin metabolism due to dysfunctional beta pancreatic cells, or peripheral resistance to it, or both (DeFronzo et al., 2015). In contrast, T1DM has an acute clinical debut in childhood, and makes the patients suffer from lack of insulin production due to chronic autoimmune destruction of beta pancreatic cells. Latent autoimmune diabetes of adults (LADA) is a sub-variation of diabetes mellitus type 1 (Djekic, Mouzeyan & Ipp, 2012), that develops in people over 30 years old (Naik, Brooks-Worrell & Palmer, 2009), and differs from classical T1DM in its gradual clinical onset (Isomaa et al., 1999).

Diabetics patients are exposed to deleterious effects of hyperglycemia throughout the years, and their risk of suffering from multiple micro and macro-vascular complications increases overtime. Multiple randomized clinical trials have shown that an intensive control of glycemic levels greatly reduces the risk of experiencing these complications (Control, of Diabetes Interventions & Group, 2005). Adequate glycemic control becomes harder to achieve as the disease advances, and increasingly complex therapies accounting for multiple comorbidities are required in patients with long standing diabetes (Longo et al., 2019). Diabetic duration is therefore a critical risk factor when managing these patients. Unfortunately, this information is sometimes unknown as the disease can progress sub-clinically for years before a diagnosis is made.

Electronic health records (EHRs) have become an integral part of medical care (Adane, Gizachew & Kendie, 2019) providing doctors with reliable information that support clinical decisions. Analysis of the accumulated data of EHRs and the implementation of predictive models is pivotal for the advancement of medicine, as it could shed a light into hidden correlations that might not be evident or clear at first sight (Štiglic et al., 2018; Benhamou, 2011). Implementation of EHRs by medical teams have improved drug treatment intensification, monitoring and physiologic control in diabetic patients (Reed et al., 2012).

Regression analysis is a widely used statistical tool in health sciences, and it is employed to illustrate the relationship between explanatory variables and a target feature (Liang & Zeger, 1993). In this context, different clinical and laboratory variables can be of use to predict past diabetes duration. Classic linear regression is often limited by non-linearity relationships, heterogeneity of effects and high dimensionality; fortunately, machine learning regression techniques have been found to overcome these limitations (Steele et al., 2018; Goldstein, Navar & Carter, 2016).

The scientific literature shows that data mining models have demonstrated to be capable of managing different facets of diabetes mellitus, in the past. For example, Bernardini et al. (2019) identified patients with early insulin resistance from health record data implementing a novel ensemble method and provided novel insights about the utilization of non-standard clinical risk factors to screen for early presentation of the disease. Machine learning techniques have predicted possible life-threatening hypoglycemic events during treatment (Georga et al., 2013), providing doctors with the capacity to tailor their treatment in this high risk population. Applied to data of EHRs of pregnant women, machine learning algorithms predicted the development of gestational diabetes, pointing out the need of a throughout screening regimen and early interventions in these patients (Artzi et al., 2020).

### Problem statement and motivation

Duration of diabetes is often unknown particularly for those patients who did not attend regular medical check-ups, and might have suffered from the disease for years before a diagnosis is made. In this group of patients, it is impossible to retrospectively know when the diabetes started. Recovering this information could be useful in foreseeing the evolution of the disease, the response to treatment, and the selection of proper screening methods (Bax et al., 2007; Pham-Short et al., 2015; Thomas, Harvey & Owens, 2016). In this context, supervised machine learning models can be used to discover past diabetes duration of the patients.

### Objective and novelty

The goal of our study is to predict the past duration of diabetes and then to detect the most predictive clinical variables. The novelty of our project lies in the usage of computational intelligence methods, together with recursive feature elimination and the coefficient of determination (R^2^) metric.

### This study

Here, our approach was first to construct a regression model on data from two different sets of health records. The diabetes type 1 dataset (Takashi2019) contains 20 variables from 73 individuals, and the diabetes type 2 (AlOlaiwi2018) contains 49 variables, from 400 patients. Our work can be described in two parts. First, we developed various regression models to predict duration of diabetes using different machine learning algorithms, resulting in Random Forests (Breiman, 2001) being our top predictor. Second, we extended our analysis by generating a ranking of key features from both datasets utilizing our best predictor (Random Forest), to unveil correlations that may be concealed from classical statistical analysis. Our ranking concluded that age, body mass index, and insulin intake are key predictors of duration of diabetes on both populations. To the best of our knowledge, no study on the prediction of past diabetes duration exists in the scientific literature.

## Datasets

For our analysis, we used two datasets, both made of electronic health records and publicly available online under the Creative Commons Attribution 4.0 International (CC BY 4.0) license: the Takashi2019 dataset of patients with diabetes type 1 (Takashi et al., 2019) and the AlOlaiwi2018 dataset of patients with diabetes type 2 (AlOlaiwi, AlHarbi & Tourkmani, 2018).

### Diabetes type 1 dataset

The Takashi2019 dataset contains data of 73 diabetic patients. Each patient profile has 20 variables, including one that indicates the past duration of diabetes in years, that we use as target variable (Table 1). The original data curators Takashi et al. (2019) collected these data at the Osaka University Hospital and Osaka Police Hospital in July and August 2017, and released them publicly in May 2019.

**Table 1:** Meaning and measurement unit of the variables of the Takashi2019 diabetes type 1 dataset. Ug/ml: microgram per milliliter. kg/m^2^ = kilogram per meter squared. pg/ml: picograms per milliliter. ml/minutes/1.73 m^2^: milliliters per minute per 1.73 m squared. m/s: meters per second. ng/ml: nanogram per milliliter.

**Feature name**	**Measurement**	**Meaning**
Added weight	kg	Calculated patient’s weight
Adiponectin	Ug/ml	Serum adiponectin
Age	Years	Age of the patient at the medical check-up
Basal	Units of insulin	Daily basal dose of insulin.
BMI	kg/m^2^	Body mass index
Bodyfat	%	Bodyfat percentage
Bolus	Units of insulin	Daily bolus dose of insulin.
Duration of diabetes	Years	Duration of diabetes type 1 from onset until the medical check-up
eGFR	ml/minutes/1.73 m^2^	Estimated glomerular filtration rate
Free-test	pg/ml	Serum free testosterone concentration
Gait speed	m/s	Walking speed on a 5 m distance
Grip strength	kg	Grip strength measured using handheld dynamometers
HbA1c	%	Percentage of glycosylated hemoglobin
Insulin regimen	binary	MDI: multiple daily injections = 1; CSII: continous subcutaneus injections = 0
Knee extension strength	kg	Knee extension strength measured using handheld dynamometers
OC	ng/ml	Total osteocalcin
Sex	Binary	male = 1; female = 0
SMI	kg/m^2^	Skeletal muscle mass index
TDD	Units of insulin	Total daily dose of insulin
ucOC	ng/ml	Undercarboxilated osteocalcin

The Takashi2019 diabetes type 1 dataset features are related to clinical characteristics of the patients (age, weight, body-mass index, sex, skeletal muscle mass index), or to her/his well-being activity (gait speed, knee extension), or to blood test results (serum adiponectin, testosterone concentration, hemoglobin, ostocalcin, underrcarboxilated osteocalcin) (Table 1).

The patients of Takashi2019 diabetes type 1 dataset have an average weight of 63.35 kg and an average age of 34.73 years (Table 2). Almost 70% of them are women and 30% are men (Table 3).

**Table 2:** Quantitative characteristics of the numeric features of the Takashi2019 diabetes type 1 dataset.

**Numeric feature**	**Median**	**Mean**	**s.d.**	**Range**
Added weight	59.40	63.35	11.91	[44.40, 104.90]
Adiponectin	12.90	14.30	6.21	[3.5, 32.3]
Age	35.00	34.73	6.16	[21, 48]
Basal	14.84	16.23	8.08	[0, 60.05]
BMI	22.87	23.76	3.47	[17.584, 35,54]
Body fat	0.26	0.27	0.07	[0.13, 0.48]
Bolus	22.88	27.63	14.96	[7.37, 93.94]
Duration of diabetes type 1 [target]	26.00	25.68	7.33	[10, 41]
eGFR	92.74	92.86	14.06	[50.7, 127.01]
Free-test	1.30	4.24	5.0	[0.4, 18.1]
Gait speed	1.31	1.34	0.22	[0.81, 2.00]
Grip strength	30.20	32.08	8.77	[16.79, 54.5]
HbA1c	7.25	7.38	1.03	[5.1, 10.7]
Knee extension strength	20.00	20.59	5.85	[8.70, 39.09]
OC	14.80	16.25	7.89	[6.4, 49.6]
SMI	6.70	6.93	0.88	[5.5, 9.2]
TDD	40.00	43.87	19.85	[15.7, 154.0]
ucOC	3.25	4.17	3.26	[0.53, 19.10]

**Notes.**

s.d. standard deviation

**Table 3:** Quantitative characteristics of the category features of the Takashi2019 diabetes type 1 dataset.

**Category feature**	**#**	**%**
Insulin regimen (0: CSII)	39	53.42
Insulin regimen (1: MDI)	34	46.58
Sex (0: female)	51	69.87
Sex (1: male)	22	30.13
Total	73	100.00

**Notes.**

# Number of patients at the medical check-up.

% Percentage of of patients at the medical check-up.

#### Diabetes type 2 dataset

The AlOlaiwi2018 diabetes type 2 dataset contains data of 400 patients from Saudi Arabia (AlOlaiwi, AlHarbi & Tourkmani, 2018). Each patient profile has 49 clinical features, including one indicating the past duration of diabetes type 2.

The original dataset curators AlOlaiwi, AlHarbi & Tourkmani (2018) collected these data at the Alwazarat Health Care Center (Riyadh, Saudi Arabia) from 1st April 2017 to 20th March 2018.

The AlOlaiwi2018 diabetes type 2 dataset consists of several features related to conditions of the patient (diabetic retinopathy, bloating, postural heart rate, vomiting, stomach fullness, belly visibly larger, gastroparesis, hypertension), physiological traits (sex, age, body-mass index), treatment (metformin, insulin, sulfonylurea), variables related to lifestyle (smoking). and laboratory test results features (eGFR, cholesterol, tryglycerides, albumn-to-creatinine ratio, hemogloblin) (Table 4).

**Table 4:** Meaning and measurement unit of the variables of the AlOlaiwi2018 diabetes type 2 dataset.

**Feature name**	**Measurement**	**Meaning**
Age	Years	Age of the patient at the medical consult
Albuminuria	Categories	Normoalbuminaria: 0, microalbuminuria: 1, macroalbuminuria: 2
Anti HTN	Binary	Taking any hipertensive drugs. 0: No 1: Yes
Bloating	Binary	Patient suffering from bloating: No: 0, Yes: 1
BMI	kg/m*2	Body mass index
CAN	Binary	Patient suffering from cardiovascular autonomic neuropathy. No: 0, Yes: 1
DBP	mmHg	Diastolic blood pressure
DDP-4 inhibitor	Binary	Prescribed DPP4 inhibitor. 0: No 1: Yes
DR	Binary	Diabetic retinopathy. 0: No, 1: Yes.
Duration of DM	Years	Duration of diabetes mellitus type 2 in years
eGFR MDRD equation	ml/min	Estimated glomerular filtration rate by the MDRD study equation
Excessive fullness after meals	Binary	Patient suffering from excessive fullness after meals: No: 0, Yes: 1
FBS	mmol/L	Fasting Blood Sugar.
GCSI category	Category	Gastroparesis cardinal symption index,
		Classified as categories: None: 0, Mild: 1, Severe: 2.
GCSI new	Point Scores	Gastroparesis cardinal sympton index score.
GCSI present ?	Binary	Gastroparesis symptomps: absent: 0, present: 1
GCSI score	Point scores	Gastroparesis cardinal symptom index score.
HbA1c	%	Percetange of glycosylated hemoglobin
HDL	mmol/L	High density lipoprotein
HTN	Binary	Hypertension: 0: No 1: Yes
Insulin	Binary	Taking insulin: 0: No 1: Yes
LDL	mmol/L	Low-density lipoprotein
Loss of appetitie	Binary	Loss of appetite for the last 2 weeks. No: 0, Yes: 1
Meglitinides	Binary	Use of Meglitinides. 0: No 1: Yes
Metformin	Binary	Use of metformin. 0: No 1: Yes
Nausea	Binary	Feelings of nausea in the last 2 weeks. No: 0, Yes: 1
None	Binary	Not taking any drug at all? 0: No 1: Yes
Not able to finish a meal	Binary	Inability to finish a regular size meal. No: 0, Yes: 1
Orthostatic hypotension	Binary	Patients suffering from orthostatic hypotension: No: 0, Yes: 1
PDBP	mmHg	Diastolic blood pressure after postural manoeuvres.
PHR	bpm	Postural heart rate
Presence of any symptom	Binary	Presence of any gastroparesis symptom: No: 0, Yes: 1
PSBP	mmHg	Systolic blood pressure after postural manoeuvres
QTc	Seconds	Corrected QT interval. (measured in the EKG)
QTc prolonged	Category	Corrected QT interval prolongation: No: 0, Borderline: 0.5 Yes: 1
Resting tachycardia	Binary	Patient suffering from resting tachycardia: No: 0, Yes: 1
Retching	Binary	Patient suffering from retching: No: 0, Yes: 1
SBP	mmHg	Systolic blood pressure
Sex	Binary	Patient’s sex: 0: female, 1: male
Smoking	Binary	Patient smoking habit: 0: No, 1: Yes
Stomach fullness	Binary	Patient suffering from stomach fullness: No: 0, Yes: 1
Stomach or belly visibly larger	Binary	Patient suffering from belly visibly larger: No: 0, Yes: 1
Sulfonylurea	Binary	Patient using sulfonylurea: 0: No 1: Yes
TC	mmol/L	Total cholesterol
TG	mmol/L	Triglycerides
TZD	Binary	Patient using thiazolidinediones: 0: No 1: Yes
UACR new	mg/g	Urine albumin-to-creatinine ratio
Urine ACR	mg/g	Urine albumin to creatinine ratio 6 months before.
Vomiting	Binary	Patient suffering from Vomiting: No: 0, Yes: 1

**Notes.**

kg/m*2 kilogram per meter squared.

mmHg millimeters of Mercury.

ml/min milliliters per minutes.

mmol/L millimole per liter.

bpm beats per minutes.

mg/g urine Albumin (mg/dL) / urine creatinine (g/dL).

This diabetes type 2 dataset contains data of patients 55.25 years old on average, with 56.25% women and 43.75% men (Tables 5 and 6).

**Table 5:** Quantitative characteristics of the category features of the AlOlaiwi2018 diabetes type 2 dataset.

**category feature**	**#**	**%**
albuminuria: macroalbuminuria	18	4.50
albuminuria: microalbuminuria	84	21.00
albuminuria: normoalbuminuria	298	74.50
anti HTN: no	143	35.75
anti HTN: yes	257	64.25
bloating: no	225	56.25
bloating: yes	175	43.75
CAN: no	339	84.75
CAN: yes	61	15.25
DDP-4 inhibitor: no	247	61.75
DDP-4 inhibitor: yes	153	38.25
DR: no	254	63.50
DR: yes	77	36.50
excessive fullness after meals: no	265	66.25
excessive fullness after meals: yes	135	33.75
GCSI category: mild	256	64.00
GCSI category: none	143	35.75
GCSI category: severe	1	0.25
GCSI present: absent	375	93.75
GCSI present: present	25	6.25
HTN: no	239	59.75
HTN: yes	161	40.25
Insulin: no	211	52.75
Insulin: yes	189	47.25
loss of appetitie: no	305	76.25
loss of appetitie: yes	95	23.75
meglitinides: no	399	99.75
meglitinides: yes	1	0.25
metformin: no	22	5.50
metformin: yes	378	94.50
nausea: no	327	81.75
nausea: yes	73	18.25
none: no	398	99.50
none: yes	2	0.50
not able to finish a meal: no	261	75.25
not able to finish a meal: yes	139	34.75
orthostatic hypothension: no	388	97.00
orthostatic hypothension: yes	12	3.00
QTc prolonged: borderline	122	30.50
QTc prolonged: no	247	61.75
QTc prolonged: yes	31	7.75
resting tachycardia: no	377	94.25
resting tachycardia: yes	23	5.75
retching: No	357	89.25
retching: Yes	43	10.75
sex Female	225	56.25
sex Male	175	43.75
smoking 0: no	359	89.75
smoking 1: yes	41	11.25
stomach fullness: no	273	68.25
stomach fullness: yes	127	31.75
stomach or belly visibly larger: no	286	71.25
stomach or belly visibly larger: yes	114	28.75
sulfonylurea: no	202	50.50
sulfonylurea: yes	198	49.50
TZD: no	397	99.25
TZD: yes	3	0.75
vomiting: no	383	95.75
vomiting: yes	17	4.25
total	400	100%

**Notes.**

# Number of patients at the medical check-up.

% Percentage of the patients at the medical check-up.

**Table 6:** Quantitative characteristics of the numeric features of the AlOlaiwi2018 diabetes type 2 dataset.

**Numeric feature**	**Median**	**Mean**	**s.d.**	**Range**
Age	55	55.25	10.646	[28, 85]
BMI	32	32.46	5.40	[17.6, 48]
DBP	74	74.52	9.52	[42, 105]
Duration of diabetes [target]	10	10.77	6.89	[0.1, 30]
eGFR MDRD equation	100.35	102.02	25.10	[42.1, 183.1]
FBS	7.7	8.71	3.55	[3.1, 25.6]
GCSI new	0.4	0.65	0.67	[0, 3.2]
GCSI score	4	5.95	6.04	[0, 29]
HbA1c	7.7	8.07	1.59	[4.8, 15]
HDL	1.12	1.15	0.34	[0.38, 3.23]
LDL	2.41	2.55	0.78	[0.99, 6.3]
PDBP	79	79.47	9.06	[55, 110]
PHR	90	79.78	13.19	[48, 136]
PSBP	132	133.95	16.09	[99, 189]
QTc	0.43	0.43	0.03	[0.36, 0.6]
SBP	130	103.32	17.08	[11, 195]
TC	4.04	4.19	0.89	[1.81, 7.96]
TG	1.52	1.70	0.81	[0.3, 7.17]
UACR new	9.155	59.92	194.49	[1.14, 2103]
Urine ACR	1.05	6.82	22.00	[0.16, 237.9]

**Notes.**

s.d. standard deviation

**Figure 1:**
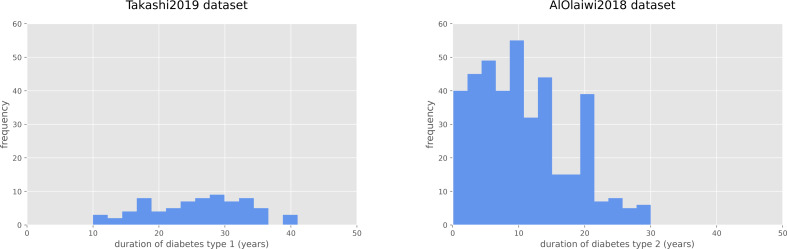
Frequency histograms of diabetes duration. Duration of diabetes type 1 for the Takashi2019 dataset (left) and diabetes type 2 for the AlOlaiwi2018 dataset (right).

The duration of diabetes type 1 for the Takashi2019 diabetes type 1 dataset patients is 25.68 years on average, and ranges between 10 and 41 years (Fig. 1). For the diabetes 2 patients of the AlOaiwi2018 dataset, instead, the duration of diabetes is 10.77 years on average, with values that range between 0.1 and 30 years (Fig. 1).

The two datasets share seven common features: age, eGFR, HbA1c, insulin intake, sex, body-mass index, and of course diabetes past duration. Additional information about the two datasets is available in the original publications (Takashi et al., 2019; AlOlaiwi, AlHarbi & Tourkmani, 2018).

## Methods

To predict the past diabetes duration for each dataset, we made a regression analysis employing several machine learning methods: Random Forests (Breiman, 2001), XGBoost (Chen & Guestrin, 2016), Linear Regression (Groß, 2012), Decision Trees (Quinlan, 1990).

We chose these data mining algorithms because they showed their strength in several biomedical informatics studies involving electronic health records in the past (Chicco & Jurman, 2020b; Chicco et al. 2023; Cerono, Melaiu & Chicco, 2023), including studies of DREAM Challenges (Meyer & Saez-Rodriguez, 2021). Moreover, tree-based machine learning algorithms are especially suitable for medical data, because they can help physicians decision-making (Podgorelec et al., 2002).

Both datasets had missing values. We addressed this problem by using the algorithm Multivariate Imputation by Chained Equations (MICE) (van Buuren & Groothuis-Oudshoorn, 2010) of the known Python package scikit-learn (Buitinck et al., 2013), under the assumptions that these values were missing at random. The MICE algorithm imputes missing data through an iterative series of predictive models utilizing other variables in the dataset.

We employed machine Learning regression algorithms directly from scikit-learn, utilizing the default values from the library for the multiple parameters available. For the regression analysis, we ran 1,000 executions with 70% randomly chosen elements for the training set and the remaining 30% for the test set (Chicco, 2017), both for regression and feature ranking through recursive feature elimination (RFE) (Darst, Malecki & Engelman, 2018).

For the diabetes past duration prediction, we employed all the variables and then saved the results measured with traditional regression rates such as the coefficient of determination (R^2^), root mean square error (RMSE), mean square error (MSE), mean absolute error (MAE), and symmetric mean absolute percentage error (SMAPE). We reported their formulas in the Supplementary Information.

For the recursive feature elimination, we repeated the tests for the numbers of features, by eliminating one feature at each run. Here we only used Random Forests, because it is the method which achieved the higher R-squared in the past diabetes duration prediction. We computed and saved the coefficient of determination for each test, and generated the ranking of the dataset features based on the increasing value of R-squared: the lower the R-squared when a specific feature is removed, the more important that feature is Chicco, Warrens & Jurman (2021). We repeated these tests 1,000 times and then merged the final rankings with the Borda’s method (Lansdowne & Woodward, 1996). The Borda’s count method consist in adding up the ranks of each variable for each iteration, resulting in a single fused ranking score after the 1,000 iterations.

This ensemble machine learning approach generated a standing of variables from tests where the features interact between each other. To further verify the importance of the datasets variables, we also produced a biostatistics ranking based on a traditional univariate test, the Kruskal–Wallis test (Kruskal & Wallis, 1952; McKight & Najab, 2010). The Kruskal–Wallis test applied to two numerical vectors of the same size generates *p*-values in the [0, 1] interval: if the two vectors are correlated, the test *p*-value is close to 0; on the contrary, if there is no correlation between the two vectors, the resulting *p*-value is close to 1. We performed this operation to see how each feature alone relates to the past diabetes duration, without interference from the other clinical variables. Following the recent biostatistics guidelines by Benjamin et al. (2018) we considered significant only the variables that obtained a *p*-value lower than 0.005, differently from 0.05 as traditionally done in the past.

## Results

In this section, we first report and describe the results obtained by the regression analysis for the prediction of the past diabetes duration (‘Prediction of the past diabetes duration’), and then we report and describe the results obtained by regression methods and biostatistics for feature ranking (‘Clinical feature ranking results’).

### Prediction of the past diabetes duration

Among the four machine learning algorithms employed for regression, Random Forests outperformed the other three methods on both the datasets, achieving an average coefficient of determination of +0.41 on the Takashi2019 diabetes type 1 dataset and an average coefficient of determination of +0.35 on the AlOlaiwi2018 diabetes type 2 dataset (Tables 7 and 8).

**Table 7:** Regression results for the prediction of the duration of diabetes type 1 on the Takashi2019 dataset. Performance of the learned models with the different methods evaluated with the different metrics, expressed in the format “average value ± standard deviation”, obtained on 1,000 executions, each execution had 70% randomly chosen data instances for training set and the remaining 30% used for test set. We reported in blue and with an asterisk * the top result for each rate. At the beginning of each execution we randomly shuffled the dataset instances. RMSE: root mean square error. MAE: mean absolute error. MSE: mean square error. SMAPE: symmetric mean absolute percentage error. R^2^: coefficient of determination. RMSE, MAE, MSE: best value 0 and worst value +∞. R^2^: best value +1 and worst value −∞. SMAPE: best value 0 and worst value 2. We listed the complete formulas of R^2^, RMSE, MSE, MAE, and SMAPE in the Supplemental Information. We ranked the methods considering the results obtained through R-squared (in bold).

**Method**	*R* ^2^	**RMSE**	**MAE**	**MSE**	**SMAPE**
Random forests	*0.41 ± 0.05	*5.98 ± 0.27	5.19 ± 0.26	*35.87 ± 03.31	0.22 ± 0.01
XGBoost	**0.39 ± 0.14**	6.04 ± 0.70	*5.00 ± 0.49	37.08 ± 08.97	*0.21 ± 0.02
Linear regression	**0.14 ± 0.47**	7.00 ± 1.83	5.52 ± 1.31	52.49 ± 29.27	0.27 ± 0.06
Decision trees	**0.05 ± 0.26**	7.53 ± 1.07	6.23 ± 0.88	57.98 ± 16.46	0.26 ± 0.03

**Table 8:** Regression results for the prediction of the duration of diabetes type 2 on the AlOlaiwi2018 dataset. These results refer to the same abbreviation meanings and execution details of Table 7 caption.

**Method**	*R* ^2^	**RMSE**	**MAE**	**MSE**	**SMAPE**
Random forests	*0.35 ± 0.02	*5.64 ± 0.11	*4.60 ± 0.10	*31.85 ± 1.30	*0.47 ± 0.01
XGBoost	**0.25 ± 0.06**	6.07 ± 0.24	4.67 ± 0.21	36.91 ± 2.98	0.49 ± 0.02
Linear regression	**0.09 ± 0.07**	6.67 ± 0.27	5.18 ± 0.21	44.54 ± 3.67	0.52 ± 0.02
Decision trees	**−0.21 ± 0.15**	7.71 ± 0.47	5.98 ± 0.39	59.69 ± 7.32	0.61 ± 0.04

**Figure 2:**
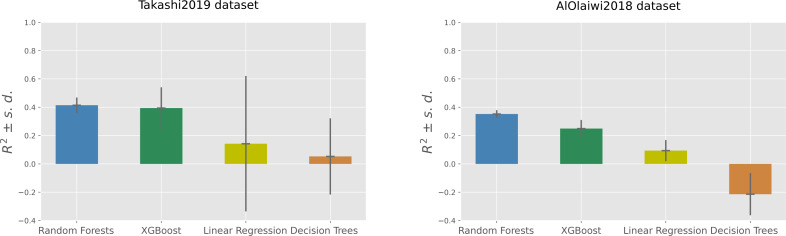
Regression results on the Takashi2019 diabetes type 1 dataset (left) and on the AlOlaiwi2018 diabetes type 2 dataset (right). Representation of the Regression results reported as mean coefficient of determination ± the corresponding standard deviations for each method. We reported the complete results measured with other rates in Tables 7 and 8.

On the diabetes type 1 dataset, Random Forests obtained the top R-squared, root mean square error, and mean square error, but was outperformed by XGBoost on the mean absolute error and on the symmetric mean absolute percentage error (Table 7). The two regression analyses generated the same standings for the results based on R-squared: Random Forests on first position, then XGBoost followed by Linear Regression, with Decision Trees on the last position (Fig. 2).

**Figure 3:**
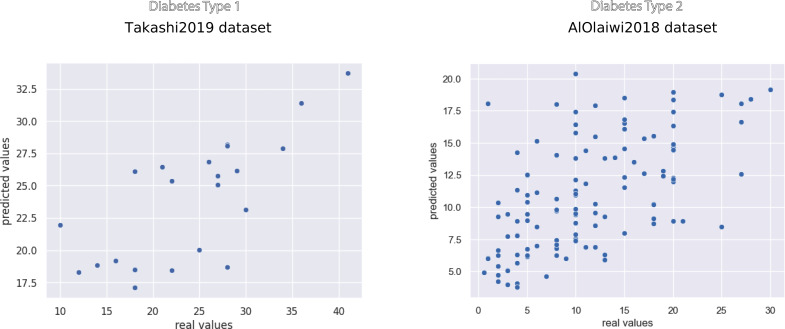
Scatterplot of the prediction results of the top methods on the Takashi2019 diabetes type 1 dataset (left) and on the AlOlaiwi2018 diabetes type 2 dataset (right). Representation of the regression results reported as actual real values *versus* predicted values, obtained through the top methods. We reported the complete results measured with other rates in Tables 7 and 8.

The scatterplots of the top performing methods (Fig. 3) shows that the majority of points is close to the *x* = *y* line, which corresponds to perfect prediction.

Regarding SMAPE, XGBoost obtained the top result of 0.21, corresponding to 89.5% correctness in the [0, 2] interval, on the diabetes type 1 dataset. Random Forests achieved the top SMAPE score of 0.47 on the diabetes type 2 dataset (Table 7), which corresponds to 76.5% correctness in the same interval (Table 8). Decision Trees obtained poor results on both dataset: an average coefficient of determination close to zero (*R*^2^ = 0.05) in Takashi2019 diabetes type 1 dataset and a negative average coefficient of determination (*R*^2^ =  − 0.21) in the AlOlaiwi2018 diabetes type 2 dataset.

### Clinical feature ranking results

The feature ranking phase based on Random Forests and recursive feature elimination (RFE) generated a standing of the datasets variables, sorted by predictive importance. On the Takashi2019 diabetes type 1 dataset, the key variables for the prediction of past diabetes duration resulted being age, daily bolus dose of insulin, and gait speed (Table 9). Among the most important variables, we also noticed estimated glomerular filtration rate (eGRF), total daily dose of insulin, grip strength, and body-mass index (BMI) (Table 9). On the bottom of the standing, the RFE put the weight of the patient, the insulin regimen, and the level of undercarboxilated osteocalcin (Table 9).

**Table 9:** Feature ranking results obtained through Random Forests on the Takashi2019 diabetes type 1 dataset. We computed the average Borda score on 1,000 executions of Random Forests. At the beginning of each execution we randomly shuffled the dataset instances.

**Rank**	**Feature**	**Average borda score**	**s.d.**
1	Age	1.275	1.719
2	Bolus	6.955	5.488
3	Gait speed	7.537	5.657
4	eGFR	8.244	5.683
5	TDD	9.683	5.467
6	Grip strength	10.517	5.116
7	BMI	10.578	5.319
8	Adiponectin	10.991	5.019
9	Basal	11.025	4.959
10	HbA1c	11.063	4.906
11	Bodyfat	11.139	4.664
12	OC	11.177	4.536
13	Sex	11.178	5.174
14	Free-test	11.200	4.798
15	Knee extension strength	11.322	4.777
16	SMI	11.458	4.521
17	ucOC	11.459	4.497
18	Insulin regimen	11.564	4.809
19	Added weight	11.635	4.535

**Notes.**

s.d. standard deviation

On the same Takashi2019 dataset, we also computed the feature ranking by using a traditional univariate statistics method: the Kruskal–Wallis test (McKight & Najab, 2010). We computed this test between each variable and the target variable (duration of diabetes type 1), and ranked the resulting *p*-values in increasing order. The results showed that no clinical variable obtained a *p*-value lower than 0.005, so no feature resulted being significant in relation with the past duration of diabetes type 1 (Table S1).

Regarding the diabetes type 2 dataset of AlOlaiwi2018, the ensemble machine learning recursive feature elimination indicated diabetic retinopathy (DR), age, insulin intake, body-mass-index, and diastolic blood pressure after postural manoeuvres (PDBP) as the top five most predictive variables for past duration of diabetes type 2 (Table 10). The same ranking indicated nausea, eGFR, and the inability to finish as the least predictive variables in the dataset (Table 10).

**Table 10:** Feature ranking results obtained through Random Forests on the AlOlaiwi2018 diabetes type 2 dataset. We computed the average Borda score on 1,000 executions of Random Forests. At the beginning of each execution we randomly shuffled the dataset instances.

**Rank**	**Feature**	**Average borda score**	**s.d.**
1	DR	3.907	8.451
2	Age	6.133	10.186
3	Insulin	6.843	9.626
4	BMI	17.256	14.731
5	PDBP	18.721	14.604
6	CAN	22.524	13.719
7	Sulfonylurea	23.417	14.038
8	HDL	23.645	13.851
9	FBS	23.927	13.910
10	LDL	24.437	13.546
11	Anti HTN	24.524	13.439
12	SBP	24.533	13.825
13	DDP-4 inhibitor	24.581	13.244
14	PHR	25.162	13.187
15	Urine ACR	25.177	12.435
16	DBP	25.241	13.712
17	QTc	25.412	13.239
18	TC	25.465	13.568
19	TG	25.681	13.450
20	HbA1c	25.742	12.959
21	Sex	25.959	12.897
22	GCSI present ?	26.133	12.506
23	UACR new	26.176	12.751
24	HTN	26.342	12.838
25	Metformin	26.542	12.599
26	PSBP	26.619	12.986
27	Resting tachycardia	26.623	12.656
28	GCSI score	26.641	12.466
29	Excessive fullness after meals	26.686	12.530
30	Vomiting	26.723	12.413
31	Meglitinides	26.830	12.307
32	Albuminuria	26.849	12.262
33	Loss of appetitie	26.850	12.532
34	Bloating	26.858	12.994
35	TZD	26.915	12.335
36	Retching	26.947	12.862
37	Stomach fullness	26.976	12.787
38	Orthostatic hypotension	27.144	12.411
39	GCSI new	27.215	12.696
40	Stomach or belly visibly larger	27.235	12.439
41	Smoking	27.242	12.754
42	Presence of any symptom	27.304	12.328
43	None	27.317	12.805
44	QTc prolonged	27.332	12.307
45	GCSI category	27.476	13.003
46	Nausea	27.546	12.334
47	eGFR MDRD equation	27.557	12.833
48	Not able to finish a meal	27.635	12.170

**Notes.**

s.d. standard deviation

The biostatistics feature ranking based on the univariate Kruskal–Wallis test found nine significant variables, which obtained *p*-values lower than the 0.005 threshold: if the patients takes no drug at all, age, insulin, diastolic blood pressure after postural manoeuvres (PDBP), diastolic blood pressure (DBP), if the patient takes in thiazolidinediones (TZD), diastolic blood pressure (PDBP), if the patient takes in metformin, and if the patient takes in sulfonylurea (Table S2). The feature indicating if the patient takes no drugs at all (none), in particular, obtained a *p*-value much lower than the other variables (2.77 × 10^−27^), which highlights its importance in the dataset.

## Discussion

In this section, we discuss the results we obtained in our scientific analyses, report some key take-home messages inferred in this study, and describe some limitations and potential future development.

### Prediction of past duration of diabetes

Our regression results on the two datasets proof that ensemble machine learning can efficiently predict the past duration of diabetes from the electronic health records of patients. The fact that our computational intelligence methods were able to obtain good results not only on one dataset but also on a second one confirms the efficacy of our approach, both on diabetes type 1 and on diabetes type 2. The Random Forests method, in particular, obtained the top results measured with the coefficient of determination both on the diabetes type 1 Takashi2019 dataset and on the diabetes type 2 AlOlaiwi2018 dataset. The gradient boosting method XGBoost, also, achieved good prediction results on both the datasets, while Linear Regression and Decision Trees did not.

These results confirm the effectiveness of ensemble machine learning and, in particular, of the Random Forests method in health informatics. Random Forests, in fact, resulted being the top performing method in multiple previous studies in this field (Chicco & Rovelli, 2019; Chicco & Jurman, 2021; Chicco & Jurman, 2020a).

Medical evidence from the scientific literature confirm the importance of diabetes past duration. Patients with long standing diabetes type 2, in fact, might have troubles controlling their glycemia (Hayashino et al., 2017). Additionally, patients who suffered diabetes for a longer time often are more in need of receiving insulin treatments, for obvious reasons (Duckworth et al., 2011).

Revealing the duration of diabetes therefore can help with the establishment of a better therapy, since a longer duration of this disease has been linked with poor glycemic control and with the consequent need of more complex medical treatment. Moreover, researchers also recorded an increase in risk of ischemic stroke in correlation with a long diabetes duration (Banerjee et al., 2012).

### Feature ranking for past duration of diabetes

As mentioned earlier (‘Datasets’), the two datasets share six common variables, in addition to past diabetes duration. Age resulted being the top most important variable in the Takashi2019 diabetes type 1 dataset feature ranking and the second most important factor in the AlOaiwi2018 dataset standing (‘Clinical feature ranking results’). This result comes with no surprise: in the medical community it is known that age is proportional to the duration of both diabetes type 1 and type 2 (Wannamethee et al., 2011; Zoungas et al., 2014).

Expectedly, insulin obtained a high ranking position on both standings (Davies et al., 2013). In the diabetes 1 dataset, the daily bolus dose of insulin taken by the patients was ranked second most important factor, while in the diabetes 2 dataset the information about the patient taking insulin or not was ranked top most relevant feature (‘Clinical feature ranking results’).

An interesting aspect of both rankings came from the positions of body-mass index in the two standings. Both the feature rankings, in fact, listed body-mass index as a top most important factor: it is found on the 7th position of the Takashi2019 diabetes type 1 dataset standing and on the 4th position of the AlOlaiwi2018 diabetes type 2 dataset standing. Several studies confirm the association between body-mass index and duration of diabetes (Bray et al., 2008; Funakoshi et al., 2008; Pencek et al., 2012).

Both the feature rankings gave average importance to HbA1c (10th position on the Takashi2019 diabetes type 1 dataset ranking and 20th position on the AlOlaiwi2018 diabetes type 2 dataset ranking), while they gave a discordant outcome for the eGFR (top position on the diabetes type 1 ranking and low position for the diabetes type 2 ranking); HbA1c is known to have an association with diabetes (Sherwani et al., 2016). Both standings listed sex as unimportant variable (13th position on the Takashi2019 diabetes type 1 dataset ranking and 21th position on the AlOlaiwi2018 diabetes type 2 dataset ranking).

These results confirm the importance of age, insulin intake, and body-mass index in the prediction of diabetes past duration from electronic health records. The role of body-mass index, especially, comes of great importance: our study results suggest that physicians and medical doctors can focus on this clinical factor to predict the past duration of diabetes, when this information is unavailable. Medical doctors can then take advantage of this inferred information for clinical decision-making, that is to decide which treatment for the patient, which screening tests, which medicines to prescribe, and all the other details.

## Conclusions

Knowing the how long a patient had diabetes is a critical information for the medical doctors to establish the correct treatment. Different durations, in fact, require different screenings, medicines, and therapies.

Even if pivotal, this information might be unavailable for patients, especially if they have just been diagnosed: since the diabetes type 2 can appear without symptoms, the diabetes diagnosis sometimes can arrive years or even decades after the diabetes onset. In these cases, a method that can calculate the past duration of diabetes in a patient from her/his clinical records can be extremely useful.

In this study, we applied several computational intelligence methods on two datasets of electronic health records of patients with diabetes (a dataset of T1DM and a dataset of T2DM) for this scope. On both the datasets, our machine learning models were able to efficiently predict the past duration of diabetes, obtaining a top average *R*^2^ = 0.41 on the Takashi2019 diabetes type 1 dataset and a top average *R*^2^ = 0.35 on the AlOaiwi2018 dataset.

After verifying the predictive efficacy of our machine learning methods for this task, we computed the feature rankings of these two datasets, through a traditional recursive feature elimination procedure. The feature ranking phase indicated age, insulin, and body-mass index as most important predictive factors on both the datasets, suggesting therefore physicians and medical doctors to focus on these elements of clinical records to foresee the duration of diabetes for any possible patient. To the best of our knowledge, no previous study utilized computational intelligence to forecast past diabetes duration and to detect the most relevant predictive variables for this scope.

Diabetic patients have increased risk of suffering from multiple and diverse diseases. Strict screening looking for early signs of pathogenesis depending on age of patients and duration of diabetes can be very useful for a correct diagnosis and prognosis. Regular diabetes type 1 usually has a sudden clinical presentation, so duration of disease is often known, but for diabetes type 2 and LADA (Latent Autoimmune Diabetes in Adults) sub variation of diabetes type 1 (Pieralice & Pozzilli, 2018; Isomaa et al., 1999), the presentation is slow and often goes misdiagnosed for years. In this context, our machine learning approach could be an effective way to retrospectively predict duration from onset.

Our computational models would allow doctors to start screening LADA patients at the right time. For example, type 1 diabetic patients generally do not develop retinopathy within 3–5 years from the diagnosis, we start screening for it with a fundoscopy after 3 years from diagnosis (Fong et al., 2004) A patient with LADA could be diagnosed 2 years late from the actual start of the disease, and therefore be 2 years late for screening as we would falsely assign a later onset.

As a limitation, we have to report that it would have been useful to have additional diabetes datasets where to verify our findings. We found other studies about analyses on electronic health records of patients with diabetes (Bächle et al., 2015; Al-Rubeaan et al., 2015; Zabeen et al., 2016; Moser et al., 2018); we contacted the corresponding authors of each of them and requested the datasets, but received no reply or our requests were rejected.

In the future, we plan to further investigate diabetes duration by analyzing data of other sources and types, such as microarray gene expression (Choi et al., 2008), RNA-Seq gene expression (Rubin et al., 2016), medical images (Samant & Agarwal, 2018), and others. We also plan to investigate data of other diseases such as heart failure (Shin et al., 2021) and amyotrophic lateral sclerosis (Kueffner et al., 2019).

## Supplemental Information

10.7717/peerj-cs.1896/supp-1 Supplemental Information 1 Change of names for dataset features, Biostatistics feature rankings, and Formulas of the regression statistical rates

## Abbreviations

ACR: albumin to creatinine ratio
BMI: body-mass index
CAN: cardiovascular autonomic neuropathy
CSII: continuous subcutaneous injections
DBP: diastolic blood pressure
DDP-4: dipeptidyl peptidase-4 inhibitor
T1DM: diabetes mellitus type 1
T2DM: diabetes mellitus type 2
DR: diabetic retinopathy
eGFR: estimated glomerular filtration rate
EHRs: electronic health records
FBS: fasting blood glucose
GCSI: gastroparesis cardinal symption index
HbA1C: percetange of glycosylated hemoglobin
HDL: high density lipoprotein
HTN: hypertension
LDL: low-density lipoprotein
MAE: mean absolute error
MDI: multiple daily injections
MSE: mean squared error
OC: osteocalcin
PDBP: postural diastolic blood pressure
PHR: postural heart rate
PSBP: postural systolic blood pressure
QTc: corrected QT interval
RFE: recursive feature elimination
RMSE: root mean square error
SBP: systolic blood pressure
SMAPE: symmetric mean absolute percentage error
SMI: skeletal muscle mass index
TC: total cholesterol
TG: triglycerides
T1D: diabetes type 1
T2D: diabetes type 2
TDD: total daily dose
TZD: thiazolidinediones
UACR: urine albumin to creatinine ratio
ucOC: undercarboxylated osteocalcin

## Additional Information and Declarations

### Data Availability

The following information was supplied regarding data availability: The Takashi2019 diabetes type 1 dataset was collected at the Osaka University Hospital and Osaka Police Hospital (Osaka, Japan) and is available at figshare: Takashi, Yuichi; Ishizu, Masashi; Mori, Hiroyasu; Miyashita, Kazuyuki; Sakamoto, Fumie; Katakami, Naoto; et al. (2019). Circulating osteocalcin as a bone-derived hormone is inversely correlated with body fat in patients with type 1 diabetes. PLOS ONE. Dataset. https://plos.figshare.com/articles/dataset/Circulating_osteocalcin_as_a_bone-derived_hormone_is_inversely_correlated_with_body_fat_in_patients_with_type_1_diabetes/8079389/1?file=15057092
The AlOlaiwi2018 diabetes type 2 dataset was collected at the Alwazarat Health Care Center (Riyadh, Saudi Arabia) and is available at figshare: AlOlaiwi, Lina A.; AlHarbi, Turki J.; Tourkmani, Ayla M. (2018). Excel sheet- data analysis dated 25 March 2018 second edition. PLOS ONE. Dataset. https://plos.figshare.com/articles/dataset/Circulating_osteocalcin_as_a_bone_derived_hormone_is_inversely_correlated_with_body_fat_in_patients_with_type_1_diabetes/8079389/1?file=15057092. Our Python software code is available under the GPL-3.0 license Github and Zenodo: - https://github.com/gabrielcerono/DiabetesColaboration
- gabrielcerono. (2023). gabrielcerono/DiabetesColaboration: publish (publish). Zenodo. https://doi.org/10.5281/zenodo.8284264
