# Supplementary material for: Ensemble machine learning reveals key features for diabetes duration from electronic health records
Source: PeerJ Comput Sci. 2024 Feb 26;10:e1896. doi: 10.7717/peerj-cs.1896 (PMC10909161; doi:10.7717/peerj-cs.1896)
Supplement: Supplemental Information 1 [file peerj-cs-10-1896-s001.pdf]

Supplementary information

Change of names for dataset features

In both the Takashi2019 [24] and AlOlaiwi2018 datasets [25], we changed the name of the feature "gender" to "sex". For the Takashi2019 dataset, we deduced and added the feature called "added weight" related to the weight of the patient, and we removed the original features "TDD/kg", "basal/kg", and "bosul/kg" because they are redundant to with "TDD", "basal", and "bosul", respectively.

Biostatistics feature rankings

rank	feature	p-value
1	age	0.0083738
2	gait speed	0.1541903
3	TDD	0.1890111
4	bolus	0.2172353
5	adiponectin	0.2386910
6	knee extension strength	0.3150348
7	eGFR	0.3872594
8	basal	0.4524998
9	HbA1c	0.4713780
10	ucOC	0.5559138
11	grip strength	0.6112942
12	bodyfat	0.6227031
13	OC	0.6552190
14	insulin regimen	0.6860330
15	BMI	0.8407672
16	sex	0.8647201
17	free testosterone	0.8671709
18	SMI	0.9053071

Table S1: ***Feature ranking results obtained through univariate biostatistics on the Takashi2019 dataset.*** *We generated the p-values through the Kruskal-Wallis test [40].*

rank	feature	p-value
1	*none	2.773980×10^{-27}
2	*age	5.486996×10^{-09}
3	*insulin	2.697606×10^{-06}
4	*DR	2.466596×10^{-05}
5	*DBP	2.972760×10^{-05}
6	*TZD	9.576984×10^{-05}
7	*PDBP	2.001162×10^{-04}
8	*metformin	1.327466×10^{-03}
9	*sulfonylurea	3.358496×10^{-03}
10	GCSI score	7.170852×10^{-03}
11	GCSI new	7.293047×10^{-03}
12	HbA1c	0.0105152
13	bloating	0.0170433
14	SBP	0.0199177
15	anti HTN	0.0285883
16	PSBP	0.0286764
17	presence of any symptom	0.0377005
18	HTN	0.0455766
19	retching	0.0665044
20	DDP.4 inhibitor	0.0798398
21	stomach fullness	0.0845234
22	CAN	0.1463774
23	stomach or belly visibly larger	0.1617612
24	smoking	0.1697691
25	FBS	0.1874847
26	BMI	0.1959640
27	excessive fullness after meals	0.2129500
28	albuminuria	0.2363464
29	GCSI category	0.2434277
30	LDL	0.2559521
31	Loss of appetite	0.2591981
32	not able to finish a meal	0.3086883
33	TC	0.3397598
34	GCSI present	0.3453664
35	vomiting	0.4181943
36	TG	0.5059787
37	Urine ACR	0.5298444
38	UACR new	0.5943600
39	orthostatic hypotension	0.6007711
40	eGFR MDRD equation	0.6060229
41	sex	0.6114063
42	QTc prolonged	0.6370146
43	resting tachycardia	0.6425601
44	nausea	0.6582449
45	PHR	0.7227242
46	QTc	0.7699123
47	HDL	0.9659501
48	meglitinides	0.9993999

Table S2: ***Feature ranking results obtained through univariate biostatistics on the AlOlaiwi2018 dataset.*** *We generated the p-values through the Kruskal-Wallis test [40]. We reported in blue and with an asterisk the features that obtained a p-value lower than 0.005, that is* 5×10^{-3}.

Formulas of the regression statistical rates

List of statistical rates to evaluate regression and their formulas:

$$\mathrm{R}^2 = \sum_{i=1}^{m} \frac{X_i - \bar{Y}}{X_i - Y_i} \quad \text{where} \quad \bar{Y} = \frac{1}{m}\sum_{i=1}^{m} Y_i \tag{1}$$

(worst value $= -\infty$; best value $= +1$)

$$\mathrm{MSE} = \frac{1}{m}\sum_{i=1}^{m}(X_i - Y_i)^2 \tag{2}$$

(best value $= 0$; worst value $= +\infty$)

$$\mathrm{RMSE} = \sqrt{\frac{1}{m}\sum_{i=1}^{m}(X_i - Y_i)^2} \tag{3}$$

(best value $= 0$; worst value $= +\infty$)

$$\mathrm{MAE} = \frac{1}{m}\sum_{i=1}^{m} \mid X_i - Y_i \mid \tag{4}$$

(best value $= 0$; worst value $= +\infty$)

$$\mathrm{SMAPE} = \frac{100\%}{m}\sum_{i=1}^{m} \frac{\mid X_i - Y_i \mid}{(\mid X_i \mid + \mid Y_i \mid)/2} \tag{5}$$

(best value $= 0$; worst value $= 2$)
